# Cardiac surgery-associated acute kidney injury: a decade of research trends and developments

**DOI:** 10.3389/fmed.2025.1572338

**Published:** 2025-04-25

**Authors:** Changlong Qiao, Jing Zhou, Chuansong Wei, Jing Cao, Ke Zheng, Meng Lv

**Affiliations:** ^1^Department of Anesthesiology, Shandong Provincial Clinical Research Center for Anesthesiology, The First Affiliated Hospital of Shandong First Medical University & Shandong Provincial Qianfoshan Hospital, Shandong Institute of Anesthesia and Respiratory Critical Medicine, Jinan, Shandong, China; ^2^Laboratory of Laparoscopic Technology, Department of Obstetrics and Gynecology, The First Affiliated Hospital of Shandong First Medical University & Shandong Provincial Qianfoshan Hospital, The First Affiliated Hospital of Shandong First Medical University, Jinan, Shandong, China; ^3^Graduate School, Shandong First Medical University & Shandong Academy of Medical Sciences, Jinan, Shandong, China; ^4^Department of Anesthesiology, Shandong Provincial Hospital of Shandong First Medical University, Jinan, Shandong, China

**Keywords:** bibliometric, acute kidney injury, cardiac surgery, VOSviewer, CiteSpace

## Abstract

**Background:**

Cardiac surgery-associated acute kidney injury (CSA-AKI) significantly increases postoperative mortality and healthcare costs. Despite the growing volume of CSA-AKI research, the field remains fragmented, with challenges in identifying high-impact studies, collaborative networks, and emerging trends. Bibliometric analysis addresses these gaps by systematically mapping knowledge structures, revealing research priorities, and guiding resource allocation for both researchers and clinicians.

**Method:**

We analyzed 4,474 CSA-AKI-related publications (2014–2023) from the Web of Science Core Collection (WoSCC) using VOSviewer, CiteSpace, the Bibliometrix Package in R, and the bibliometric online analysis platform.

**Results:**

Annual publications increased steadily, with the USA and China leading productivity. The *Journal of Cardiothoracic and Vascular Anesthesia* serves as the foremost preferred journal within this domain. *Critical Care* (IF = 15.1) has the highest impact factor. Yunjie Li published the most papers. John A Kellum has the highest H-index. The definition, pathogenesis or etiology, diagnosis, prediction, prevention and treatment, which are the research basis in CSA-AKI. Machine learning (ML) and prediction models emerged as dominant frontiers (2021–2023), reflecting a shift toward personalized risk stratification and real-time perioperative decision-making. These advancements align with clinical demands for early AKI detection and precision prevention.

**Conclusion:**

This study not only maps the evolution of CSA-AKI research but also identifies priority areas for innovation: multicenter validation of predictive models to strengthen generalizability, preventive nephrology frameworks for long-term AKI survivor monitoring, and randomized controlled trials to confirm efficacy of machine learning-based CSA-AKI prediction tools.

## Introduction

Acute kidney injury (AKI) is a major postoperative complication affecting millions of cardiac surgery patients worldwide each year (1, 2). Among intensive care patients, CSA-AKI ranks as the second most common cause of AKI and has shown a rising incidence in recent years (3). Clinically, CSA-AKI significantly increases mortality risks and extends ICU hospitalization (4), making it a critical focus in perioperative care. The study of CSA-AKI has gained significant attention from researchers across the worldwide. Many studies related to CSA-AKI have been reported over the past few decades, making it very difficult for scholars to identify highly influential articles and research hotspots.

Currently, bibliometric analysis helps researchers identify research priorities, trends, and emerging hotspots in a field (5, 6). Moreover, co-citation is commonly utilized in bibliometric analysis. The visualized form of co-citation analysis in this context can simplify data interpretation and render the results more exhaustive (7). During the analysis, details including authors, journals, keywords, institutions, references, countries/regions, citations and textual information can be collected. Therefore, the development of a discipline or research field can be achieved through bibliometric analysis (8). Bibliometrics, serving as a supplementary research approach, has gained extensive application across numerous disciplines (9). However, few bibliometric studies on CSA-AKI have been reported. This bibliometric analysis was to spot the most impactful literature, emerging trends, and research hotspots to provide a comprehensive overview of the current status of CSA-AKI.

## Materials and methods

### Data source and collection

**Figure 1:**
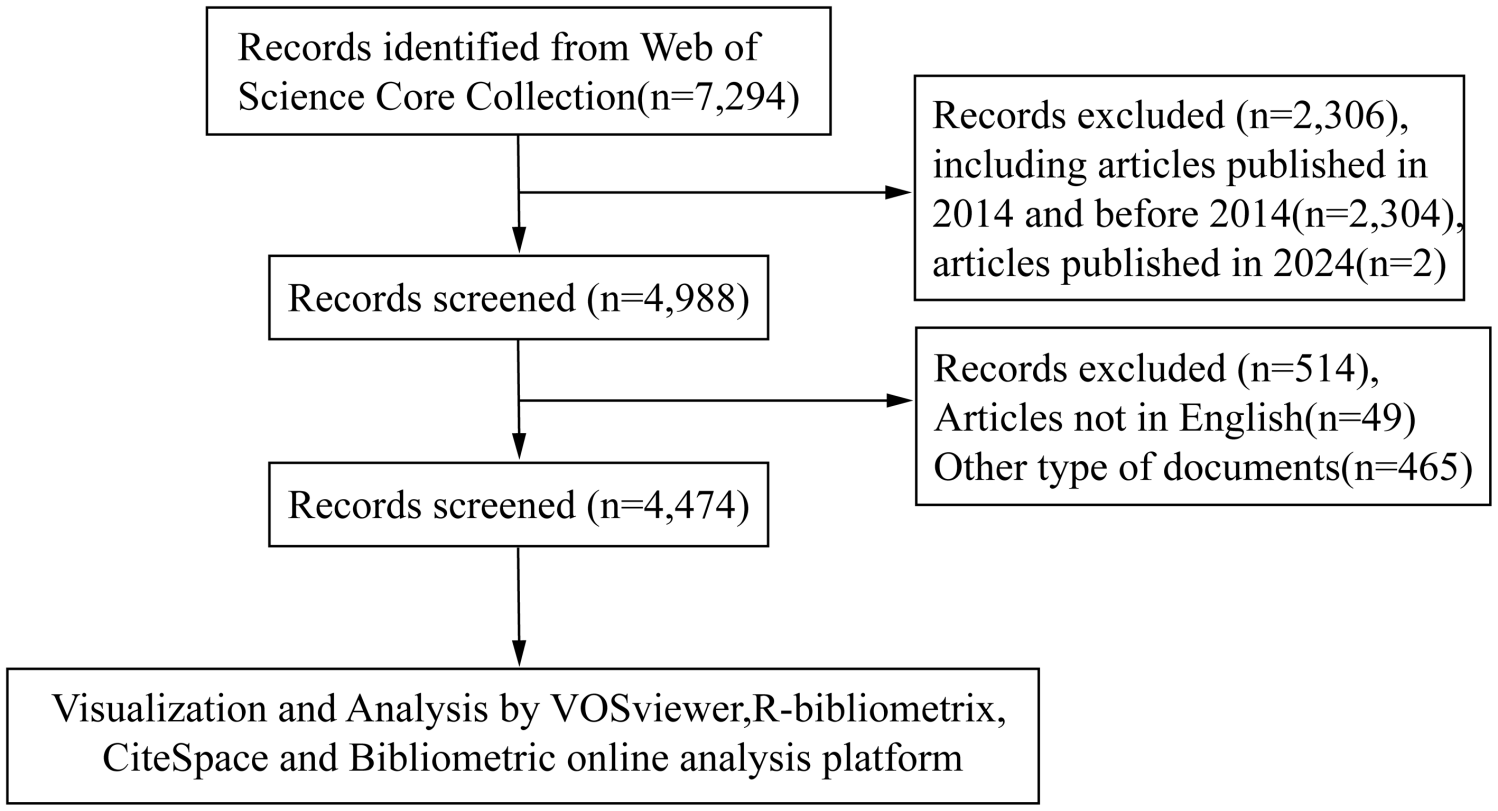
Data retrieval flowchart.

The WoSCC, a premier and authoritative research database, encompasses comprehensive scholarly publications across diverse disciplines (10). Recognized as an optimal resource for bibliometric studies, it has gained widespread adoption within the academic community due to its rigorous curation standards and multidisciplinary coverage (11). WoSCC offers comprehensive citation metrics and compatibility with bibliometric tools, enabling systematic analysis of co-authorship, co-citation, and keyword trends. While PubMed is a critical resource for biomedical research, its limited citation metrics and lack of compatibility with bibliometric tools (e.g., inability to export full citation networks) made it less suitable for our analytical framework. Similarly, although Scopus offers broader coverage in engineering and clinical domains, its citation data structure differs from WoSCC, complicating cross-tool integration (12). WoSCC provides granular metadata (e.g., cited references, institutional affiliations) essential for visualizing collaboration networks and intellectual bases. Furthermore, WoSCC’s selective indexing ensures high-quality publications, reducing noise from non-peer-reviewed sources (13). These features align with our study’s goal of mapping influential works and trends in CSA-AKI research. Therefore, the data were only obtained from the WoSCC database. To guarantee comprehensive and precise data retrieval, the indexes were selected as SCI-Expended. We standardized the search strategy before searching for articles. To ensure comprehensive and unbiased literature retrieval, we standardized search terms using Medical Subject Headings (MeSH) from the U.S. National Library of Medicine. Core concepts were mapped to their official MeSH terms using the PubMed MeSH database. For example: *“Acute kidney injury”* → MeSH: “Acute Kidney Injury.” *“Cardiac surgery”* → MeSH: “Cardiac Surgical Procedures.” To account for variant terminology, we included all synonyms and free-text variations of the MeSH terms. For instance: *MeSH term “Acute Kidney Injury”* was expanded to: (“acute renal failure” OR “acute renal insufficiency” OR “acute kidney failure”). *MeSH term “Cardiac Surgical Procedures”* included: (“heart surgery” OR “surgery, cardiac” OR “surgery, heart”). This approach minimized bias from inconsistent terminology and ensured maximal retrieval of relevant publications. The full search strategy, including MeSH terms and free-text variations, is provided in Supplementary material. The timespan was 2014–2023. To minimize the bias resulting from frequent database updates, we carried out all literature retrieval and data downloads within one day, Jan 3, 2024. A total of 7,294 publications were identified from the WoSCC. To ensure transparency and reproducibility, the following criteria were applied: Inclusion Criteria: Study Types: Original research articles and review articles; Topic: Studies explicitly addressing CSA-AKI; Timeframe: Articles published between January 1, 2014, and December 31, 2023. Exclusion Criteria: Non-English articles; Duplicate records. The retrieved publications were exported with “full records and cited references in plain text format.” Records exported from WoSCC were imported into EndNote (X9.3.3) for duplicate screening. Using title similarity (≥90%) and author/year matching criteria, no duplicates were identified, confirming the uniqueness of the 4,474 retained records. (Figure 1).

### Data analysis

We imported WoSCC data into VOSviewer 1.6.18 (Leiden University, Leiden, The Netherlands), CiteSpace V6.1.R2 (64-bit) Basic (Drexel University, Philadelphia, PA, US), the Bibliometrix 4.1 package based on the R language, and the bibliometric online analysis platform^1^ were used to perform the bibliometric analysis.

VOSviewer was used to construct and visualize bibliometric maps for co-authorship, co-citation, bibliographic coupling, and co-occurrence analyses, including network visualization, overlay visualization and density visualization. Its clustering algorithms excel at identifying thematic groups and collaborative patterns. The program provides a viewer, enabling users to examine bibliometric maps meticulously (14).

CiteSpace was applied to detect emerging trends in a research field and render them visually. A specialty is visualized and conceptualized as a time-variant duality between research fronts and intellectual bases. The intellectual base is determined through an algorithmic process, taking into account the time factor, by means of newly emerging research-front terms. The significance of a co-citation cluster is clearly explained using research front concepts. Pivotal points detected through algorithms effectively simplify the complexity of a visualized network. This enables a clear illustration of the knowledge evolution process and the time-span of documents within a cluster, allowing for a better understanding of the development process and trends in this area of study (15).

The bibliometric online analysis platform and the bibliometrix package based on the R language are also used for data processing and visualization (16). Bibliometrix, provides comprehensive statistical analyses (e.g., annual publication trends, country/institution productivity) and integrates seamlessly with R for advanced data manipulation. Its strength lies in generating descriptive metrics and cross-tabulating metadata.

By combining these tools, we ensured a multi-dimensional perspective: VOSviewer captured spatial and relational patterns (e.g., collaboration networks). CiteSpace revealed temporal dynamics and intellectual turning points (e.g., citation bursts). Bibliometrix provided granular statistical validation and metadata synthesis. This triangulation minimized methodological biases and enriched the interpretability of results, as no single tool could address all aspects of a complex bibliometric inquiry.

## Results

### Annual growth trend of publications

**Figure 2:**
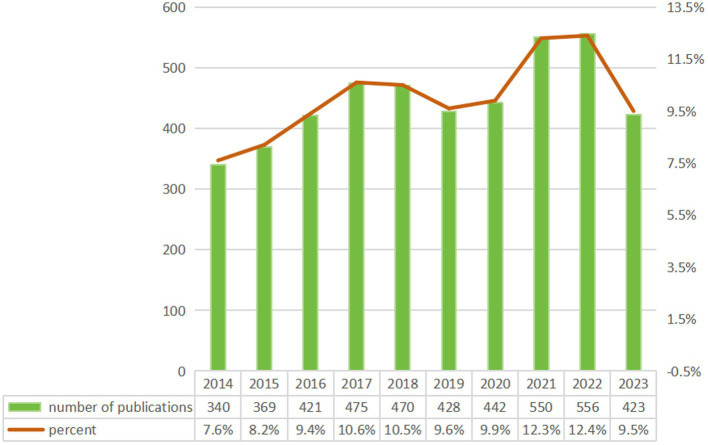
Annual publication growth trend.

Our analysis identified 4,474 CSA-AKI related publications (2014–2023) from 776 journals in the WoSCC, comprising 3,786 (84.6%) original articles and 688 (15.4%) reviews. These works involved 21,406 authors affiliated with 17,394 institutions globally. As shown in Figure 2, annual publications exhibited steady growth over the decade – from 340 in 2014 to 569 in 2022 (67% increase). This marked rise reflects expanding research engagement with CSA-AKI, underscoring its growing clinical and scientific importance.

### Distribution of publications by country and institution

**Figure 3:**
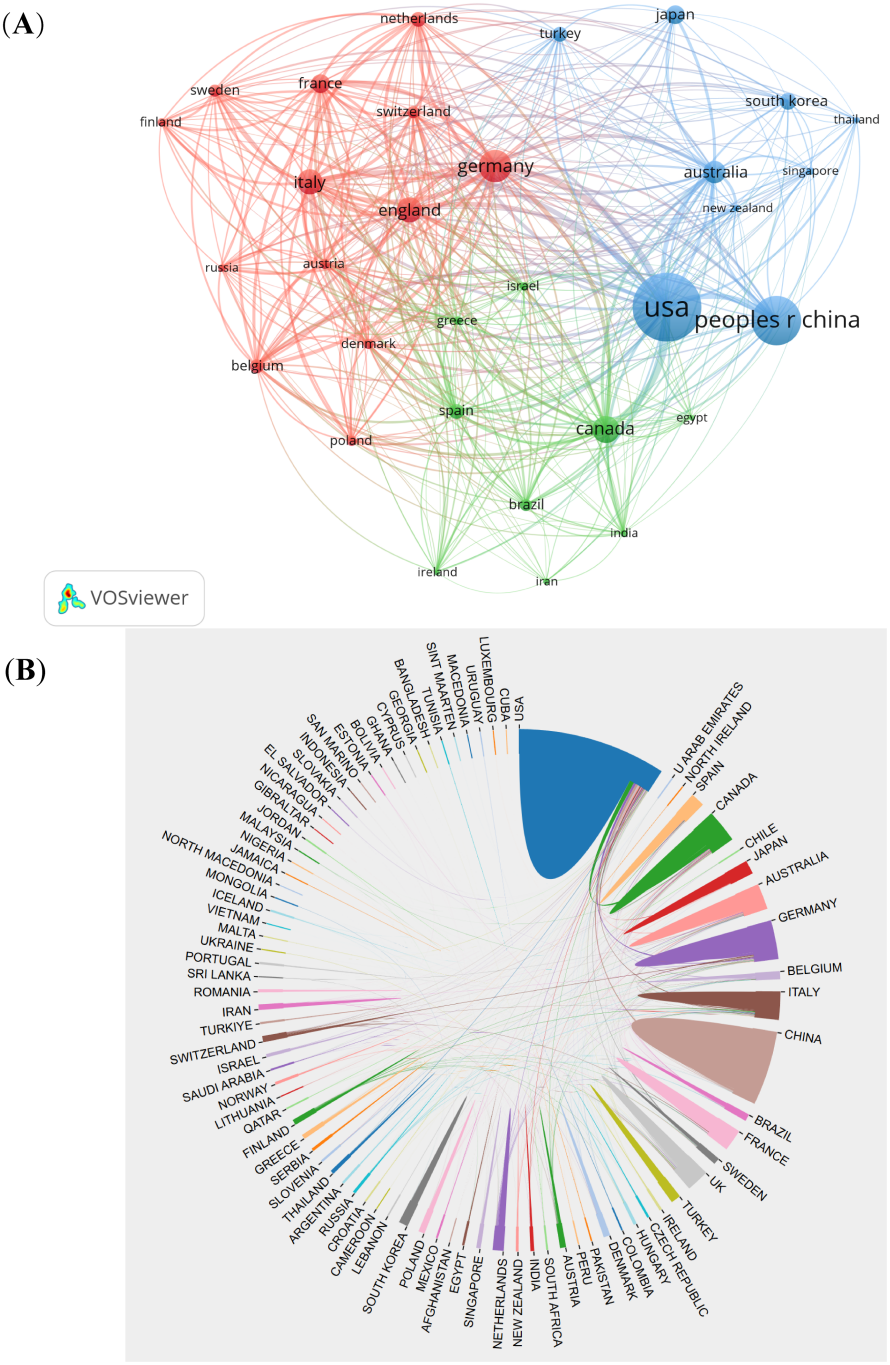
The visualization of countries **(A)** and cooperation between countries **(B)** on research of CSA-AKI.

A total of 94 countries/regions contributed to the 4,474 CSA-AKI publications analyzed. The top 10 countries accounted for 76.6% of global output, led by the United States (1,212 publications, 27.1%), China (825, 18.4%), Germany (316, 7.1%), Italy (183, 4.1%), Canada (173, 3.9%), the United Kingdom (167, 3.7%), Japan (150, 3.4%), Korea (144, 3.2%), Australia (142, 3.2%), and France (113, 2.5%). International collaboration patterns were visualized through co-authorship analysis of 38 countries with ≥20 publications (Figure 3A). Key findings include: The United States served as the central hub, maintaining strong partnerships with China, Germany, and Australia. China demonstrated intensive collaborations with the U.S., Canada, and Italy. A tightly connected cluster emerged among the U.S., China, Germany, Italy, Australia, the UK, and France (Figure 3B). This collaborative network underscores the globalized nature of CSA-AKI research, with major contributors actively sharing knowledge across geographic boundaries.

**Figure 4:**
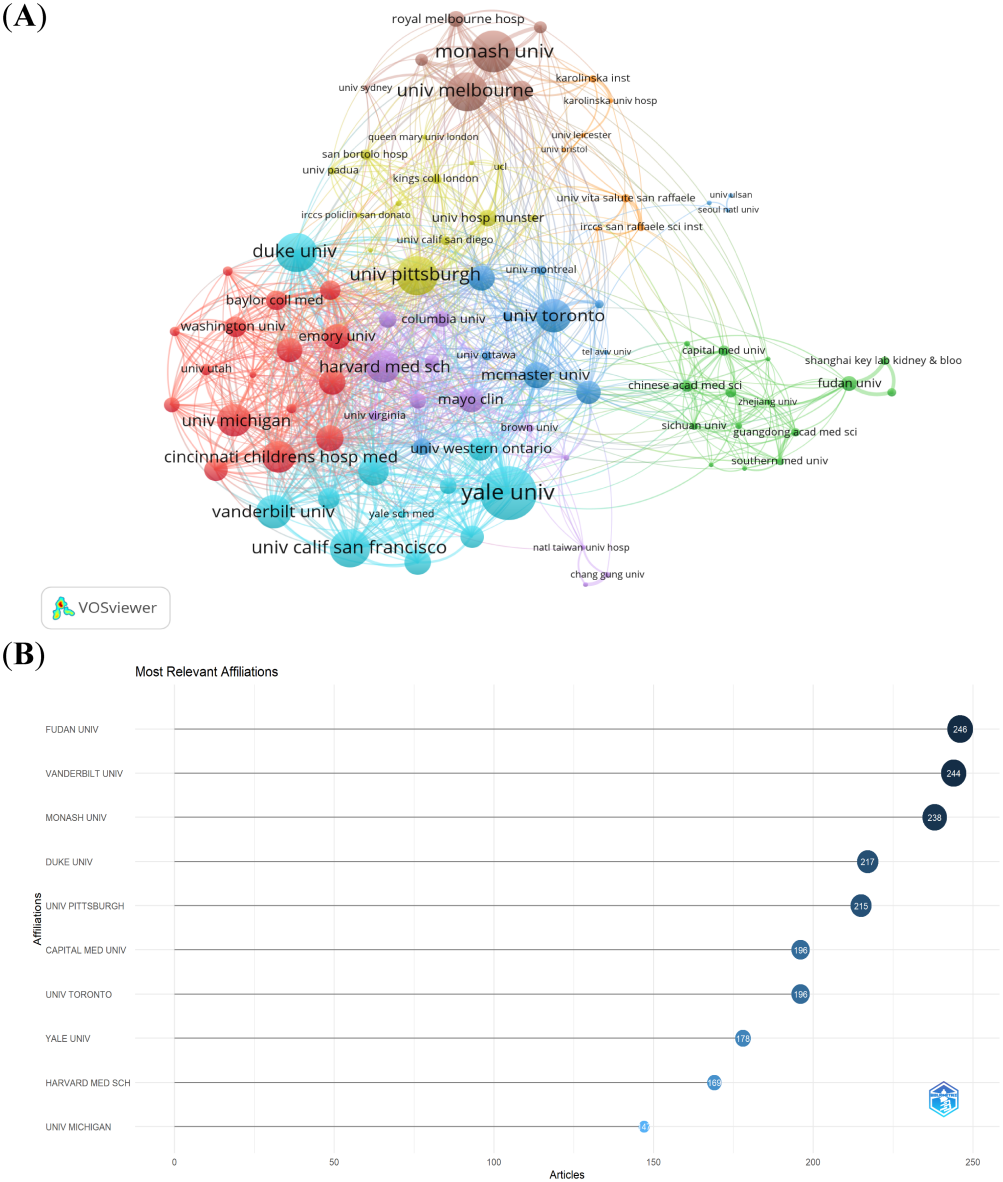
The visualization of institutions **(A)** and the most relevant affiliations **(B)** on research of CSA-AKI.

Using a threshold of ≥20 publications, we analyzed collaboration networks among 94 institutions (Figure 4A). Key metrics included publication volume, co-authorship frequency, and total link strength. Yale University (USA) emerged as the most connected hub (highest total link strength). Monash University, University of Melbourne (Australia), and Duke University (USA) ranked next in collaboration centrality. 60% of top 10 collaborative institutions were U.S.-based, confirming its leadership. Other leading institutions spanned China, Australia, and Canada. Fudan University (China) led in publication count (Figure 4B). Six of the top 10 productive institutions were U.S. affiliates. This analysis reveals a U.S.-centric collaborative network with growing contributions from Chinese institutions.

### Journals and co-cited journals

**Figure 5:**
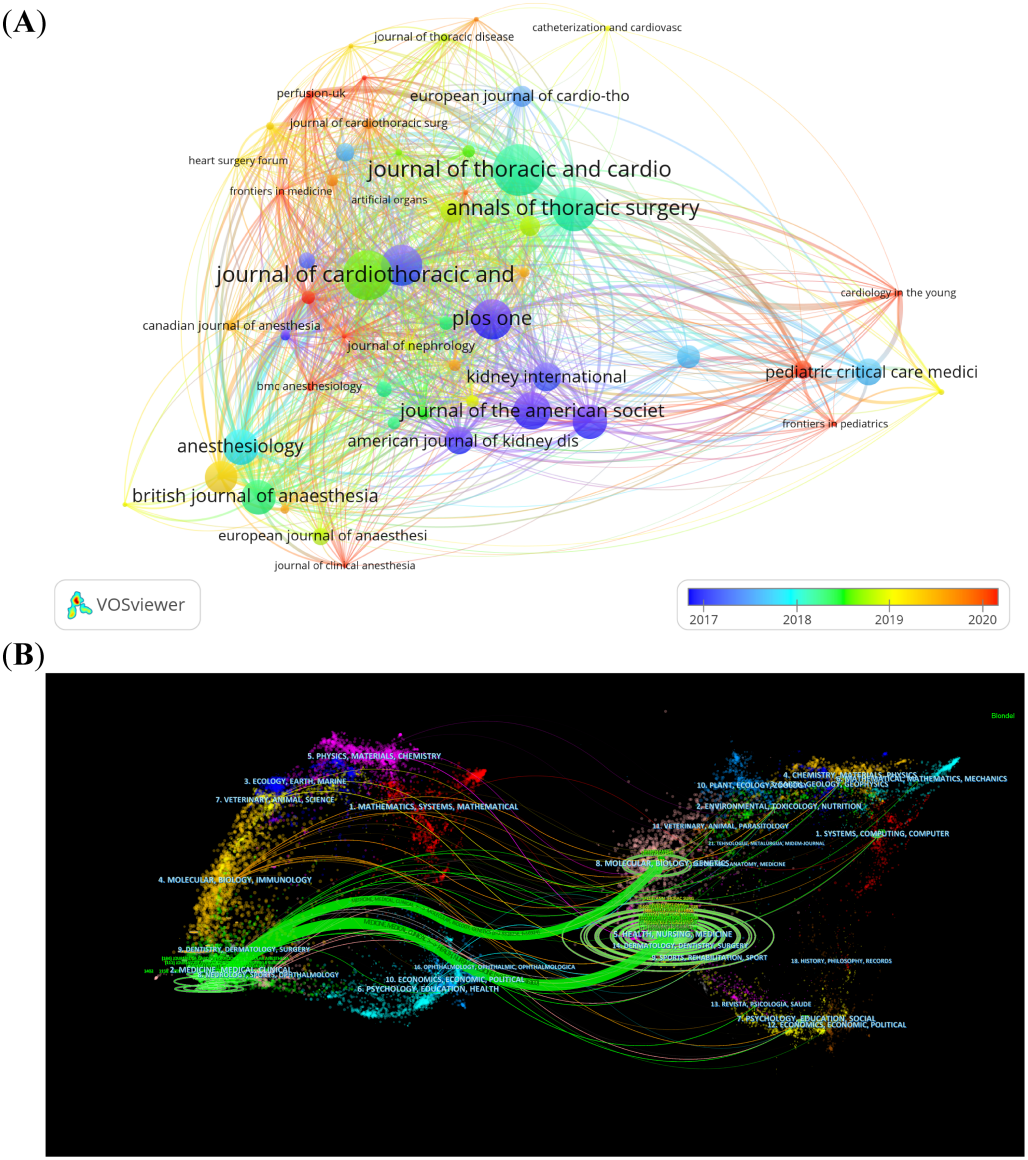
The visualization of journals **(A)** and dual-map overlay of journals **(B)** on research of CSA-AKI.

Throughout the past decade (2014–2023), 776 different journals have put out articles on the topic of CSA-AKI. The *Journal of Cardiothoracic and Vascular Anesthesia* published the most papers (*n* = 184, 4.1%), followed by the *Annals of Thoracic Surgery* (*n* = 119, 2.7%), the *Journal of Thoracic and Cardiovascular Surgery* (*n* = 111, 2.5%) and *Plos One* (*n* = 80, 1.9%). Of the top 10 journals, *Critical Care* had the highest impact factor (IF = 15.1), followed by the *Journal of Thoracic Cardiovascular Surgery* (IF = 6) and *Anesthesia and Analgesia* (IF = 5.9). Based on the JCR 2022 standards, the three journals described earlier are classified into the Q1 category and their impact factors exceed five. Based on a minimum of 20 publications, we screened 55 journals for visualization. Then, we mapped the journal network by considering the number of publications, their relationships, and the citations of each journal (Figure 5A). Figure 5A shows that the *Journal of Thoracic and Cardiovascular Surgery* has active citation relationships with *Anesthesiology*, *British Journal of Anaesthesia* and *Anesthesia & Analgesia*, etc. The dual-map overlay of journals depicts the citation relationships between journals and their co-cited counterparts. The cluster of citing journals is on the left, while the cluster of cited journals is on the right (17). Figure 5B shows two main citation paths. The articles that were published were predominantly centered around the medicine, medical, and clinical domains. The majority of the cited articles were printed in journals within the areas of molecular biology, genetics, as well as health, nursing, and medicine. This pattern reflects CSA-AKI’s evolution from basic science discoveries to clinical applications.

### Authors and co-cited authors

**Figure 6:**
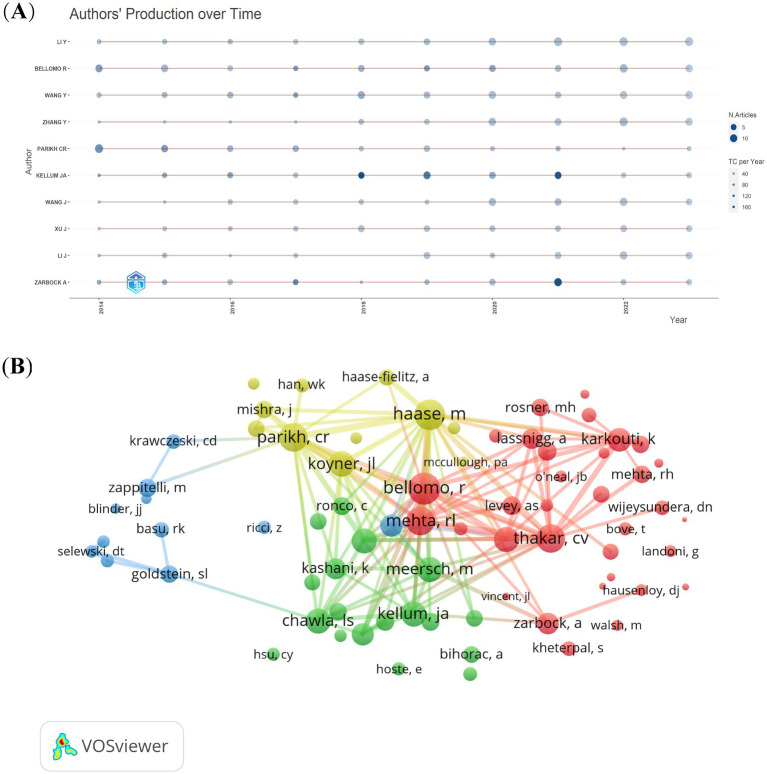
The authors’ production over time **(A)** and co-cited authors **(B)** on research of CSA-AKI.

From the 4,474 publications, a total of 21,406 related authors were listed. The results regarding the top 10 authors having the highest number of publications are presented in Figure 6A. The red line represents the author’s timeline. The size of the bubbles corresponds to the number of publications. Specifically, the larger the bubble, the greater the number of publications by the author in that particular year. The intensity of the bubble color indicates the total number of citations of the publications in that year. The darker the color, the higher the total number of citations for the author’s publications in that year. Rinaldo Bellomo is the most relevant author and has published 71 articles in the CSA-AKI field, followed by Yiou Wang (69) and Chirag R Parikh (18). Based on the H-index and the number of publications, we determined the 10 most influential authors in the CSA-AKI field (Table 1). John A Kellum is the most influential author and had the highest H-index and total number of citations.

**Table 1:** Top 10 most influential authors in the CSA-AKI.

Rank	Author	Institution (Affiliation)	Article counts	Total citations	Average number of citations	H-index
1	Kellum JA	University of Pittsburgh, USA	49	4,007	81.78	28
2	Parikh CR	Johns Hopkins School of Medicine, USA	50	2097	41.94	28
3	Bellomo R	Monash University, Australia	71	2,221	31.28	22
4	Garg AX	Western University, Canada	34	1,306	38.41	22
5	Ronco C	San Bortolo Hospital, Vicenza, Italy	34	1811	53.26	19
6	Wang Y	The George Institute for Global Health, Australia	69	1,344	19.48	18
7	Zarbock A	University Hospital Münster, Germany	42	2,406	57.29	18
8	Koyner JL	The University of Chicago, USA	25	1,006	40.24	16
9	Ranucci M	IRCCS Policlinico San Donato, Milan, Italy	25	1,013	40.52	16
10	Devarajan P	Cincinnati Children’s Hospital Medical Center, USA	26	803	30.88	15

From the 59,759 co-cited authors, we filtered out those with a minimum of 150 co-citations and then mapped a co-citation network graph (Figure 6B). As shown in Figure 6B, the most co-cited author is Rinaldo Bellomo. Active collaborations also exist among various co-cited authors. Examples include the partnerships between Rinaldo Bellomo and Ravindra L Mehta, as well as between Chirag R Parikh and Michael Haase.

### Co-cited references

**Figure 7:**
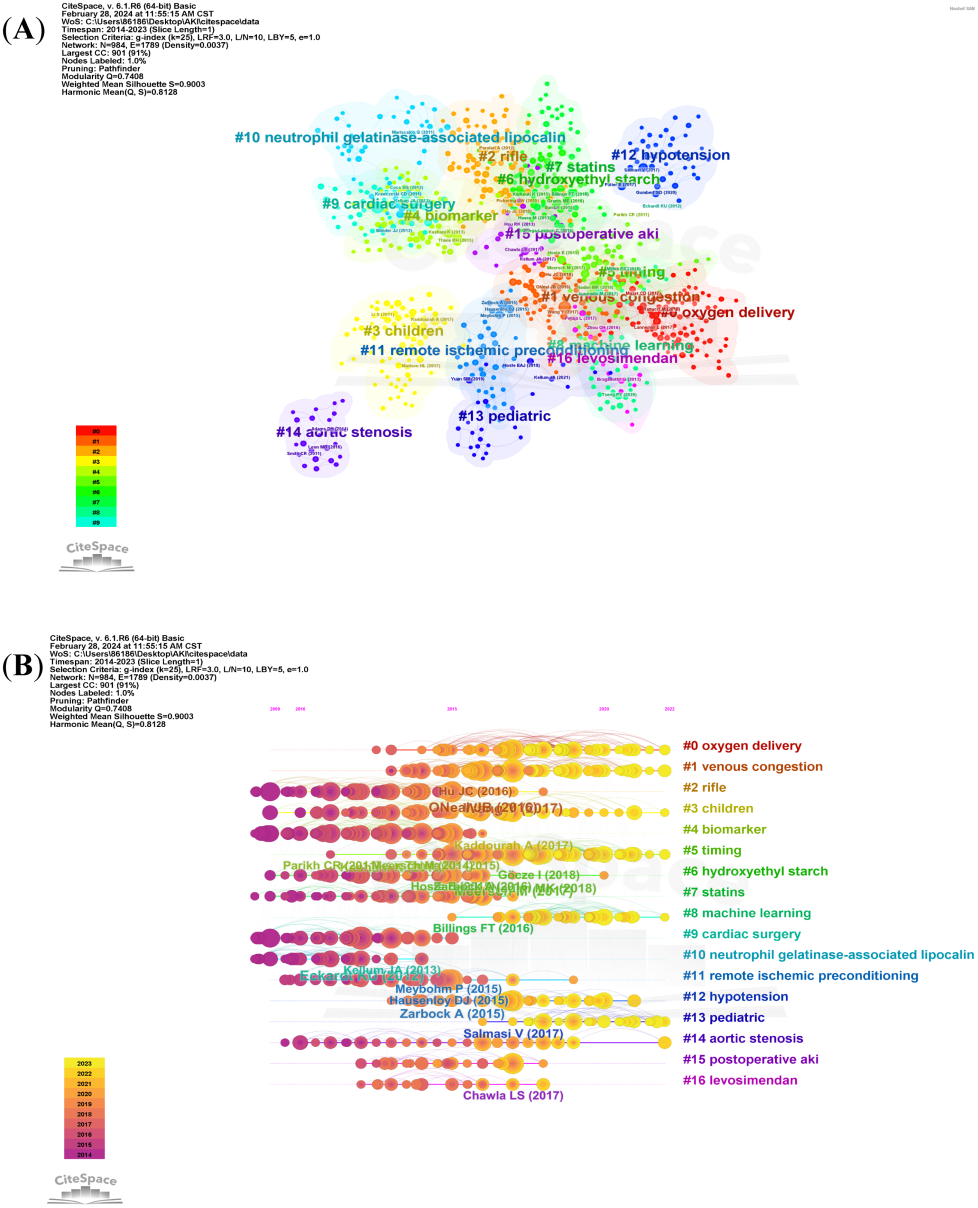
The reference co-citation and cluster network **(A)** and co-citation literature timeline map **(B)** on research of CSA-AKI.

Over the past decade, a total of 94,109 co-cited references have emerged in the research of CSA-AKI. In the list of the top 10 co-cited references presented in Table 2, each reference has been co-cited at least 200 times. Notably, one of these references has been co-cited more than 600 times (19). We generated co-citation and clusters network maps via CiteSpace (Figure 7A). The reference co-citation and cluster network contained 984 nodes and 1789 links. Within this network, every node stands for a referenced article, and the size of the node is in proportion to the total co-citation frequency of the corresponding article. As depicted in Figure 7A, the co-cited references were grouped into 16 primary cluster labels: oxygen delivery, venous congestion, RIFLE, children, biomarker, timing, hydroxyethyl starch, statins, machine learning, cardiac surgery, neutrophil gelatinase-associated lipocalin, remote ischemic preconditioning, hypotension, pediatric, aortic stenosis, postoperative AKI and levosimendan. Each cluster is presented as a horizontal timeline running from left to right, as shown in Figure 7B. The co-citation literature timeline map indicates that the concept of oxygen delivery (#0) experienced the most significant citation bursts. Moreover, it appears that the focal point of research has been evolving. Initially centered around aspects such as cardiac surgery (#9), neutrophil gelatinase – associated lipocalin (#10), and biomarker (#4), it has now shifted toward oxygen delivery (#0), venous congestion (#1), machine learning (#8), and pediatric (#13) related aspects.

**Table 2:** Top 10 co-cited references.

Rank	Reference	Citations	Key findings	Relevance to CSA-AKI research
1	Mehta RL et al. (19)	619	Proposed the (AKIN) criteria, standardizing AKI diagnosis and staging.	Standardized AKI definitions, enabling consistent reporting and risk stratification.
2	Eckardt KU (41)	511	Proposed a framework for evidence-based guidelines and pinpointed critical research gaps.	The recommendations are crucial for advancing CSA-AKI research and improving clinical outcomes.
3	Bellomo R et al. (20)	465	Established a framework for defining, classifying, and managing ARF, applicable to CSA-AKI research.	Standardized definitions, outcomes, fluid protocols, and IT strengthened CSA-AKI research rigor.
4	Rosner MH et al. (42)	366	Summarizes CSA-AKI epidemiology, mechanisms, prevention, and unmet research needs.	Critical to CSA-AKI research for risk stratification, mechanism elucidation, and intervention development.
5	Thakar CV et al. (43)	339	A validated 17-point score predicts post-cardiac surgery ARF using key risk factors.	It enhances risk prediction for CSA-AKI, improves clinical utility, and supports clinical trials and early interventions.
6	Hobson CE et al. (44)	334	Links CSA-AKI to higher long-term mortality, even with mild AKI or renal recovery.	Highlights the critical need for early detection, risk stratification, and long-term follow-up in CSA-AKI patients.
7	Khwaja A (21).	296	The KDIGO guidelines provided a comprehensive framework for managing AKI, including CSA-AKI.	Guidelines for CSA-AKI prevention, treatment, and monitoring improve evidence-based care in cardiothoracic surgery.
8	Lassnigg A et al. (45)	286	Revealed the prognostic significance of early postoperative serum creatinine changes.	Supports early AKI detection, risk stratification, and management in post-cardiac surgery CSA-AKI patients.
9	Karkouti K et al. (46)	239	Preoperative anemia correction, RBC reduction, and reexploration avoidance may lower CSA-AKI risk.	It offered potential avenues for reducing the incidence and improving the outcomes of CSA-AKI.
10	Chertow GM et al. (47)	230	AKI severity directly correlates with increased mortality, LOS, and costs.	Reveals AKI’s economic/public health burden, urging targeted interventions for high-risk groups.

### Reference with citation bursts

**Figure 8:**
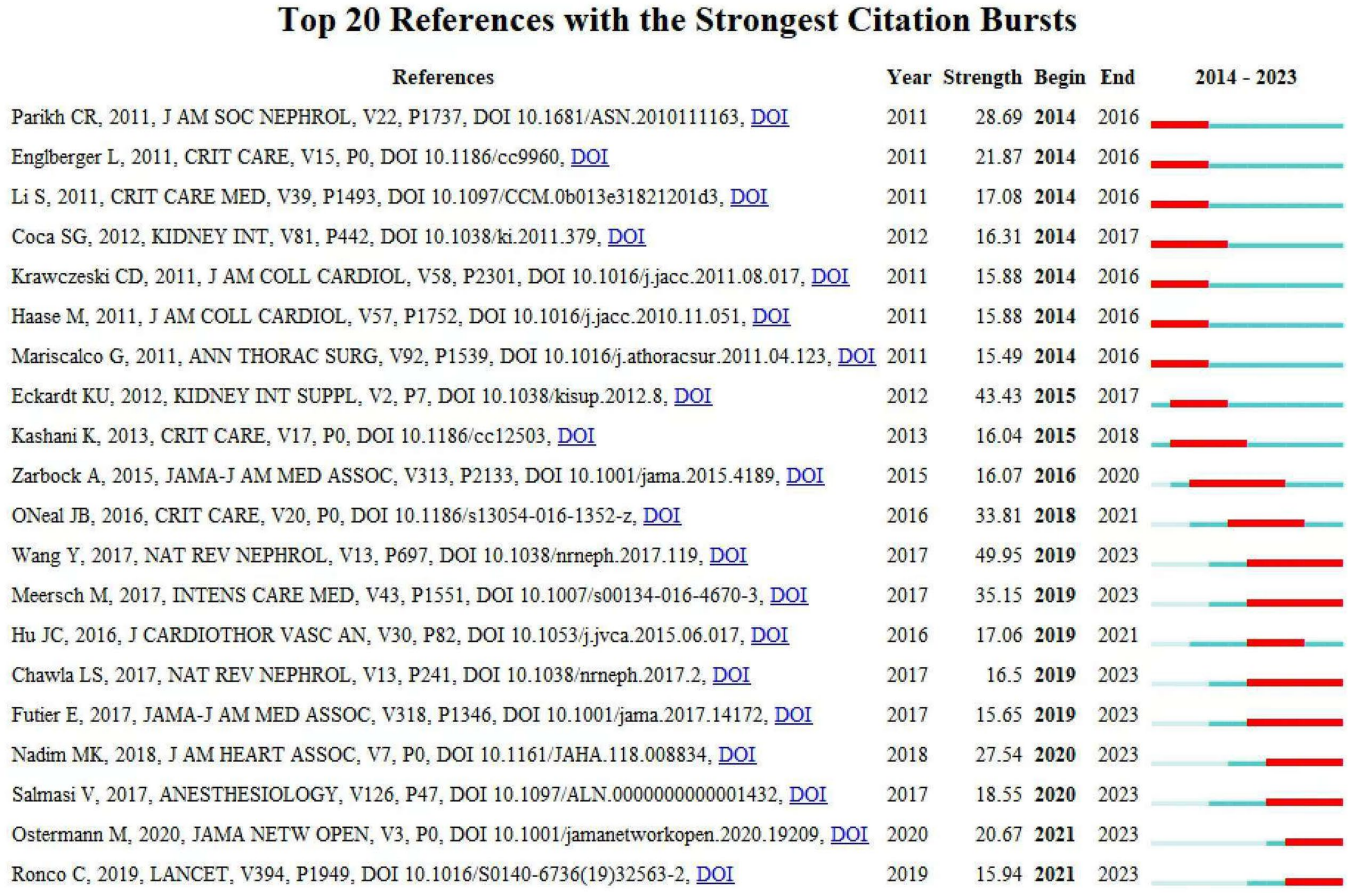
Top 20 references with strong citation bursts. A red bar indicates high citations in that year.

References with citation bursts are defined as those that are frequently cited by scholars within a particular field over a specific period. In our research, we used CiteSpace to identify 20 references exhibiting strong citation bursts (Figure 8). In Figure 8, the lines represent the timeline, the red line segments represent strong citation burstiness by showing the burst’s start year, end year and duration. These bursts correlate with three distinct phases of evolution:

*Early Phase (2014–2017): Standardization of Definitions and Biomarker Discovery*–*Key Bursts*: References Mehta et al. (19), Bellomo et al. (20), and Khwaja et al. (21). These works established standardized AKI diagnostic criteria (e.g., AKIN, KDIGO) and validated biomarkers (e.g., NGAL, cystatin C). For instance, Mehta et al.’s AKIN criteria (burst strength = 28.69) unified AKI staging, enabling consistent epidemiological reporting and risk stratification. This standardization catalyzed biomarker-driven research, as seen in Haase et al. (22), which linked NGAL to subclinical AKI detection. Transitioned the field from descriptive observational studies to mechanistic investigations of CSA-AKI pathophysiology.

*Middle Phase (2018–2020): Mechanistic Insights and Preventive Strategies–Key Bursts*: References Zarbock et al. (23), Meersch et al. (24), and Futier et al. (22). Zarbock et al.’s trial (burst = 16.07) demonstrated the efficacy of remote ischemic preconditioning (RIPC), sparking global interest in non-pharmacological renoprotection. Similarly, Futier et al. highlighted personalized hemodynamic management, shifting clinical paradigms toward proactive intraoperative optimization. Emphasis on modifiable risk factors (e.g., anemia, hypotension) and preventive interventions (e.g., RIPC, KDIGO bundles), reducing reliance on reactive post-AKI therapies.

*Recent Phase (2021–2023): Machine Learning and Precision Prevention–Key Bursts*: References Wang et al. (4), Ostermann et al. (25), and Luo et al. (26). Wang et al.’s (4) review (burst = 49.95) synthesized pathophysiology and risk stratification frameworks, providing a foundation for ML-driven prediction models. Ostermann et al. (25) (burst = 20.67) established consensus criteria for biomarker use in acute kidney injury (AKI), which provided a clinical foundation for risk stratification and early intervention strategies. Subsequent studies, such as Luo et al. (26), demonstrated the integration of these biomarkers into machine learning models to predict AKI in specific clinical contexts (e.g., pediatric cardiac surgery). These works reflect a paradigm shift toward real-time, personalized risk prediction using dynamic variables (e.g., intraoperative hypotension, biomarkers). Transition from generalized protocols to precision medicine, leveraging ML to integrate multi-modal data (e.g., biomarkers, hemodynamics) for early AKI detection and prevention.

The citation bursts highlight a dynamic evolution from foundational definitions (2004–2012) to biomarker-driven prevention (2015–2020) and ML-enabled precision medicine (2021–2023). Each burst represents a conceptual or methodological leap, addressing prior limitations (e.g., diagnostic inconsistency, reactive management) and redirecting research toward actionable, personalized strategies. Table 3 presents a summary of the key research contents of the top 20 references, following the order depicted in Figure 8.

**Table 3:** The main research contents of the 20 references with strong citations bursts.

Rank	Strength	Main research content
1	28.69	Biomarkers measured after surgery can predict the occurrence of acute kidney injury and unfavorable outcomes following pediatric cardiac surgery (55).
2	21.87	The clinical validity of the RIFLE and Acute Kidney Injury Network criteria in diagnosing acute kidney injury among patients who have cardiac surgery (56).
3	17.08	The prevalence, seriousness, and risk determinants of acute kidney injury in kids undergoing cardiac operations for congenital heart anomalies (57).
4	16.31	AKI serves as an independent risk factor for mortality and other significant non-renal outcomes (58).
5	15.88	The temporal association and predictive significance of urinary acute kidney injury biomarkers following pediatric cardiopulmonary bypass (59).
6	15.88	When there is no diagnostically significant rise in serum creatinine, neutrophil gelatinase-associated lipocalin can identify patients who probably have subclinical AKI and are at an elevated risk of adverse outcomes (60).
7	15.49	A great deal more research is required for the prevention and management of AKI after cardiac operations (61).
8	43.43	The GRADE system was utilized to assess the strength of evidence and the strength of recommendations (41).
9	16.04	Urinary insulin-like growth factor-binding protein 7 and tissue inhibitor of metalloproteinases-2 offer supplementary details beyond clinical variables and bring in mechanistic understanding regarding AKI (62).
10	16.07	Impact of remote ischemic preconditioning on renal injury in high-risk patients undergoing cardiac surgery (23).
11	33.81	Current attempts to decrease AKI after cardiac surgery are mainly limited to hemodynamic adjustments and strict monitoring of intravenous resuscitation strategies (31).
12	49.95	Acute kidney injury associated with cardiac surgery: risk factors, pathophysiological mechanisms, and therapeutic approaches (4).
13	35.15	The prevention of cardiac surgery-associated AKI through the application of KDIGO guidelines in high-risk patients identified by biomarkers (24).
14	17.06	AKI places a significant burden on patients undergoing cardiac surgery and has the potential to influence both their short-term and long-term prognoses (63).
15	16.5	The Acute Disease Quality Initiative puts forward definitions, staging criteria for acute kidney disease, and strategies for managing the patients who are affected (64).
16	15.65	The influence of personalized versus standard blood pressure management approaches on postoperative organ impairment among high-risk patients during major surgeries (22).
17	27.54	Conduct a review of the existing literature regarding AKI associated with cardiac and vascular surgery, so as to provide suggestions for clinical practice and put forward a framework for future research (38).
18	18.55	The association between intraoperative hypotension, which is defined either by a decrease from the baseline or by absolute thresholds, and acute kidney as well as myocardial injury following non-cardiac surgery (52).
19	20.67	Based on the existing data and expert consensus, formulate recommendations regarding AKI biomarkers for practicing clinicians and researchers (25).
20	15.94	AKI has evolved in definition, understanding, and management. New biomarkers and techniques have emerged. There are still controversies, and some regions lag in addressing AKI (65).

### Hotspots and frontiers

By conducting a co-occurrence analysis of keywords, we can rapidly identify the key trends and hotspots within specific fields. Table 4 presents the top 20 high-frequency keywords in research of CSA-AKI. Among these, keywords such as cardiac surgery, mortality, acute kidney injury, outcomes, risk, acute renal failure, risk factors, surgery and cardiopulmonary bypass appeared more than 400 times. These keywords signified the primary research directions within the realm of CSA-AKI.

**Table 4:** Top 20 keywords on research of CSA-AKI.

Rank	Keywords	Occurrences
1	Cardiac-surgery	1,423↑
2	Mortality	1,067↑
3	Acute kidney injury	1,027↑
4	Outcomes	943↑
5	Risk	733
6	Acute-renal-failure	723↑
7	Risk-factors	494↑
8	Surgery	462↑
9	Cardiopulmonary bypass	441↑
10	Disease	399
11	Impact	348
12	Management	332
13	Biomarkers	308↑
14	Failure	288
15	Association	263
16	Dysfunction	260
17	Gelatinase-associated lipocalin	247
18	Therapy	232
19	Critically-ill patients	223
20	Injury	223↑

↑ indicates keywords that have increased in prominence over time.

**Figure 9:**
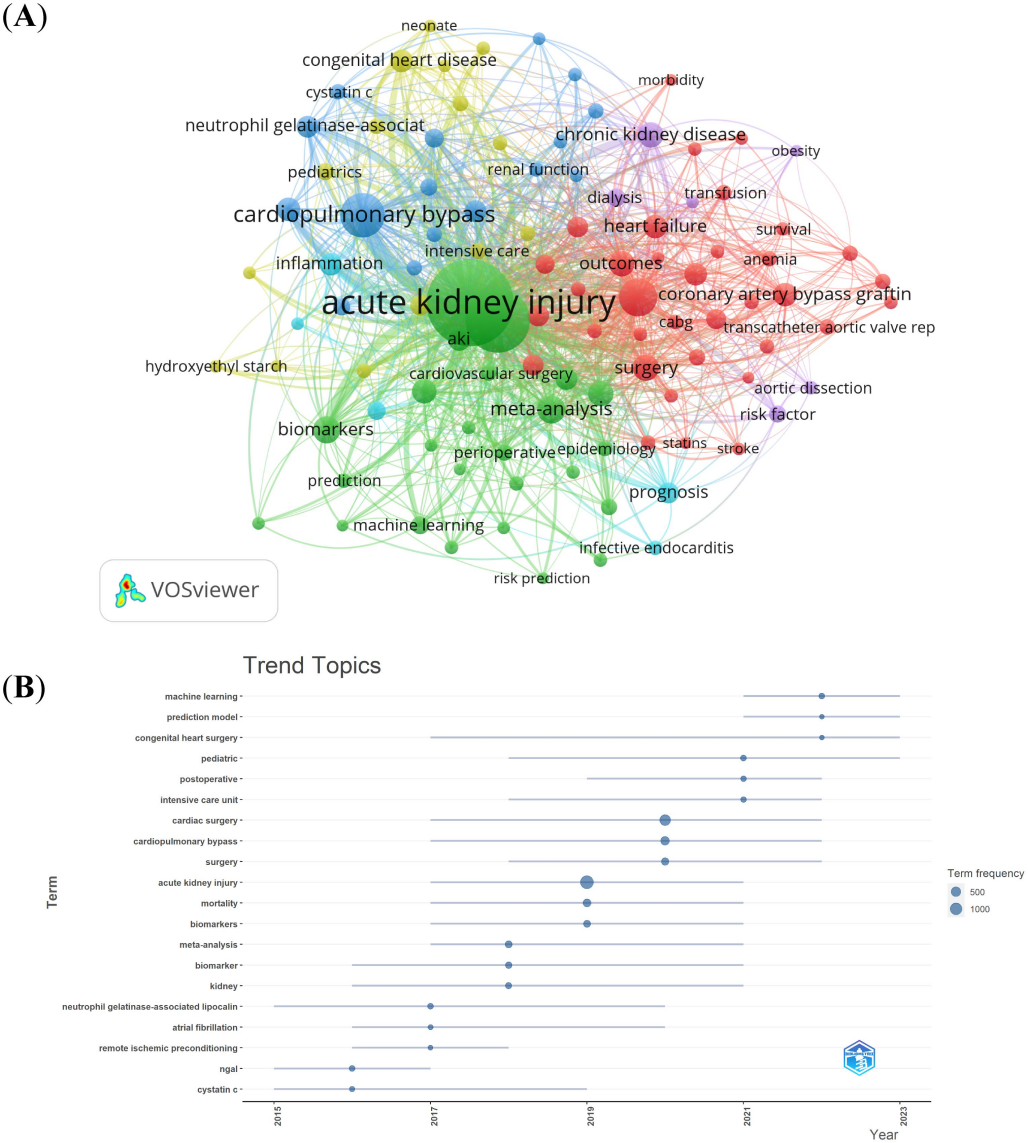
Keyword cluster analysis **(A)** and trend topic analysis **(B)**.

Out of the 5,564 keywords, we filtered those with a minimum occurrence count of more than 20. Subsequently, we carried out a cluster analysis using VOSviewer (Figure 9A). As shown in Figure 9A, we obtained 6 clusters, representing 6 research directions. Cluster 1 (red) centers around the pathogenesis as studied in basic research; cluster 2 (green) is primarily concerned with clinical research aimed at exploring the prognosis and treatment of patients suffering from kidney injury; cluster 3 (blue) relates to the epidemiological characteristics of CSA-AKI; cluster 4 (yellow) focused on diagnostic and treatment methods; cluster 5 (purple) focused on risk factors relate to CSA-AKI; cluster 6 (blueness) relates to prognosis of CSA-AKI. The trend topic analysis of the keywords, as depicted in Figure 9B, indicated that until 2017, research during this time predominantly concentrated on basic research. The aim was to explore the pathogenesis of CSA – AKI, and the primary keywords included cystatin c, neutrophil gelatinase-associated lipocalin, remote ischemic preconditioning, atrial fibrillation, etc. Starting from 2017, scholars initiated their exploration into the pathogenesis, prognosis, and treatment aspects within the domain of CSA-AKI, and the main keywords were biomarker, acute kidney injury, cardiac surgery, intensive care unit, postoperative, prediction model, etc. Moreover, over the past three-year period from 2021 to 2023, machine learning and prediction models emerged as dominant frontiers, reflecting a shift toward personalized risk stratification and real-time perioperative decision-making. These advancements align with clinical demands for early AKI detection and precision prevention.

## Discussion

In this study, we summarized research hotspots and collaborations and developed research trends for CSA-AKI by using the WoSCC database, VOSviewer, CiteSpace, Bibliometrix Package based on the R language and bibliometric online analysis platform. Distinct from previous bibliometric analyses that either focused on broader AKI contexts (e.g., sepsis, chronic kidney disease) without cardiac surgery specificity (27, 28) or relied on single analytical tools, our multi-modal methodology enables robust visualization of collaborative networks and thematic evolution. By analyzing journals and co-cited references, we recognized the most productive authors and institutions, and we illustrated the collaboration networks with different research groups. The analysis reveals three key research domains: (1) diagnostic biomarkers (cystatin C, neutrophil gelatinase-associated lipocalin), (2) surgical interventions (cardiovascular bypass optimization, remote ischemic preconditioning), and (3) emerging predictive analytics, particularly machine learning-driven risk stratification models (2021–2023). While early co-cited references focused on AKI diagnostic criteria (29, 30), recent citation bursts emphasize risk stratification and personalized care bundles (1, 26), reflecting the field’s transition from phenomenological observation to actionable predictive analytics in CSA-AKI management.

### General information

We identified a total of 4,474 articles written by 21,406 authors from 17,394 institutions, which were published in 776 journals. The annual number of CSA-AKI publications increased steadily from 2014 to 2023, reflecting growing scholarly engagement with this critical complication. This trend likely stems from multiple interrelated factors: *Increased Clinical Awareness*: The adoption of standardized diagnostic criteria over the past decade has facilitated more consistent identification and reporting of CSA-AKI cases, prompting further investigation into its epidemiology, mechanisms, and outcomes (19, 24). *Technological Advancements*: The emergence of novel biomarkers (e.g., NGAL, cystatin C) and machine learning (ML) prediction models has revolutionized risk stratification and early diagnosis (26). *Therapeutic Innovation*: Recent studies emphasize modifiable risk factors (e.g., hemodynamic optimization, avoidance of nephrotoxic agents) and preventive strategies (e.g., remote ischemic preconditioning), fostering a paradigm shift from reactive to proactive management (23, 26).

The United States and China are key countries engaged in CSA-AKI research. Among the top 10 research institutions, 60% are based in the United States. International research cooperation is a crucial element in advancing technological innovation and research progress. We found closer cooperation among these countries: USA, China, Germany, Italy, Australia, UK and France. Collaborations among different countries are vibrant. The United States actively engages in cooperation with China, Germany, and Australia. Meanwhile, China has intensive partnerships with the United States, Canada, and Italy. Yale University exhibits the highest total link strength. Monash University, the University of Melbourne, and Duke University follow it in that order. Nevertheless, our findings also indicate that Fudan University has made the largest contribution in terms of the number of publications. Nevertheless, these institutions seldom engage in cooperation with other ones, and this situation impedes the long-term development of academic research. Even though there are certain collaborative ties with some countries, both the intensity and scope of such cooperation fall short of expectations. Consequently, we suggest that the cooperation and communication among research institutions ought to be strengthened to advance the development of CSA-AKI.

Among the top 10 journals, *Critical Care* has emerged as a pivotal platform for advancing CSA-AKI research, bridging foundational discoveries with clinical innovation. With the highest impact factor (IF = 15.1) among top CSA-AKI journals, it consistently publishes high-impact studies that redefine diagnostic criteria, therapeutic strategies, and methodological frameworks. The 2016 review by O’Neal et al. (31) synthesized pathophysiological mechanisms (e.g., ischemia–reperfusion injury, inflammation) and preventive strategies (e.g., hemodynamic optimization, cardiopulmonary bypass minimization). This work highlighted the unmet need for early AKI biomarkers and personalized interventions, directly influencing subsequent clinical trials and guideline updates. *Critical Care* has been instrumental in promoting technological innovation. The 2020 study by Tseng et al. (32) demonstrated the superiority of machine learning models (Random Forest AUC = 0.84) over traditional scoring systems in predicting CSA-AKI. By integrating intraoperative hemodynamic data and biomarkers, this work exemplified the journal’s role in fostering AI-driven precision medicine. Such studies have catalyzed a paradigm shift toward real-time risk prediction, aligning with the keyword trends identified in our analysis (Figure 9B). *Critical Care* has profoundly shaped CSA-AKI research by publishing transformative studies that blend mechanistic rigor with clinical applicability. Its influence extends beyond citation metrics–by fostering consensus guidelines, promoting AI-driven innovations, and bridging gaps between basic science and perioperative care, the journal remains a cornerstone of modern CSA-AKI scholarship. Future efforts should leverage its platform to validate predictive models in multicenter cohorts and address long-term outcomes in AKI survivors, as highlighted in our bibliometric priority areas.

The *Journal of Cardiothoracic and Vascular Anesthesia* (JCVA) has emerged as a pivotal platform for disseminating CSA-AKI research, publishing 184 articles (4.1% of global output) between 2014–2023. JCVA has consistently highlighted innovations in CSA-AKI risk stratification. For instance, Tian et al. (33) developed a prediction score for postoperative AKI in Chinese populations, integrating dynamic intraoperative variables like hypotension and fluid balance, which has informed personalized monitoring protocols. Similarly, Silverton et al. (34) explored urinary biomarkers (e.g., TIMP-2, IGFBP7) for real-time AKI detection, demonstrating their prognostic value in guiding KDIGO bundle implementation. These studies exemplify JCVA’s emphasis on translating biomarker research into clinical decision-making tools. JCVA has prioritized studies on modifiable intraoperative risks. Mitrev et al. (35) identified elevated preoperative pulse pressure as a strong predictor of AKI severity, advocating for stricter hemodynamic control during cardiopulmonary bypass. Additionally, Heringlake et al. (36) investigated the nephrotoxic effects of gelatin-based fluids, prompting shifts toward balanced crystalloid use in volume resuscitation. Such work underscores JCVA’s role in refining perioperative guidelines. While *Critical Care* leads in mechanistic studies, JCVA distinguishes itself through clinical pragmatism. For instance, Milne et al. (37) provided actionable recommendations for high-risk CSA-AKI patients, contrasting with *Critical Care*’s focus on biomarker discovery. JCVA has shaped CSA-AKI research by prioritizing clinically relevant studies on prediction, prevention, and personalized management. Its emphasis on multidisciplinary collaboration and real-world applicability cements its status as a cornerstone journal for anesthesiologists and surgeons navigating the complexities of AKI in cardiac surgery. Future editions should continue fostering innovation in predictive analytics and multicenter trials to address unresolved challenges in renal protection.

John A Kellum is the most influential author and had the highest H-index and total number of citations. As a lead author of the 20th ADQI Consensus Conference (2017), he established standardized diagnostic criteria and risk stratification frameworks, reshaping clinical paradigms (38). His epidemiological studies, including foundational work on CSA-AKI pathophysiology, elucidated incidence rates, modifiable risks, and cardiopulmonary bypass-related renal ischemia–reperfusion injury mechanisms (39). Kellum’s biomarker-driven innovation was exemplified in the PrevAKI trial, showing that urinary TIMP-2 and IGFBP7 guided KDIGO protocols reduced AKI incidence in high-risk patients, now a cornerstone of precision prevention (3). Rinaldo Bellomo is the most relevant author and has published 71 articles in the CSA-AKI field, followed by Yiou Wang and Chirag R Parikh. Rinaldo Bellomo, the most co-cited author in this analysis, has significantly advanced understanding of CSA-AKI pathophysiology and therapeutic strategies. As a lead author of seminal reviews, he systematically characterized CSA-AKI epidemiology and identifying modifiable risk factors. His work established the pathophysiological framework linking renal hypoperfusion, neurohormonal activation, and inflammation to tubular injury, which remains foundational to current mechanistic models (4). His methodological innovations extend to therapeutic trials. The randomized trial, co-led by Bellomo, demonstrated that perioperative intravenous amino acid infusion reduced AKI incidence, mechanistically attributed to improved renal perfusion and oxygen delivery. This trial redefined renoprotective strategies, shifting paradigms from reactive to proactive hemodynamic and metabolic support (40). In addition, there exist dynamic and active collaborative efforts among the different co-cited authors, such as Rinaldo Bellomo and Ravindra L Mehta, Chirag R Parikh and Michael Haase.

### Knowledge base

Co-cited references indicate the frequency with which two publications are cited concurrently by other works. As such, they can be regarded as the foundation of research within a specific field (15). In the course of this bibliometric research, the top 10 co-cited references were chosen in order to establish the research foundation of CSA-AKI. Ravindra L Mehta et al. had the most co-cited study published in 2007. This research put forward unified criteria for the diagnosis and classification of AKI based on the existing system (19). The second co-cited publication was published in *Kidney International Supplements* by Kai-Uwe Eckardt et al., who provided comprehensive evidence-based recommendations on the basis of the GRADE system (41). In 2004, Rinaldo Bellomo et al. published the third co-cited paper in *Critical Care* (20). This research explored the definitions of acute renal failure, the means of measuring outcomes, animal models, fluid therapy, and the requirements in information technology. The *Clinical Journal of the American Society of Nephrology* published the fourth co-cited study by Mitchell H Rosner et al. in 2006 (42). This prospective research showed that the mechanisms underlying kidney injury during cardiopulmonary bypass include hemodynamic, inflammatory, and other aspects. Moreover, no pharmacological interventions have been proven effective in preventing renal impairment following cardiac surgery. The fifth co-cited publication was published in 2005 by Charuhas V Thakar et al., and this study provided a clinical score that predicts acute renal failure after open-heart surgery (43). The sixth co-cited study was published in *Circulation* by Charles E Hobson et al., and this research disclosed that, over a 10 – year period, the mortality risk linked to AKI following cardiothoracic surgery stays elevated, even among patients who experience full renal recovery (44). The seventh co-cited study was published in 2012 by Arif Khwaja. The focus of these guidelines is on the AKI definition, the prevention and treatment of AKI, contrast-induced AKI, as well as dialysis interventions for treating AKI (21). In 2004, the eighth most commonly co-cited paper was published by Andrea Lassnigg et al. in *Journal of the American Society of Nephrology*. They discovered that even a slight rise in serum creatinine is correlated with an elevated mortality rate. For any patient in whom such minor changes in renal function become apparent, clinicians need to be vigilant and prevent anything that could further damage renal function (45). Keyvan Karkouti et al. analyzed the modifiable risk factors of CSA-AKI in *Circulation* and published the ninth co-cited paper. They stated that treatments focused on reducing preoperative anemia and limiting perioperative red blood cell transfusions might provide protection from CSA-AKI (46). The last co-cited paper was published in the *Journal of the American Society of Nephrology*. This study suggested that there is a significant association between AKI and an increased mortality rate. From a public-health standpoint, non-dialysis-requiring AKI might be of equal or even greater significance. Consequently, preventing and effectively treating hospital-acquired AKI ought to be a national priority (47). Overall, the top 10 co-cited papers focused on the following topics: definition, pathogenesis or etiology, diagnosis, prediction, prevention, and treatment.

### Emerging topics

References with citation bursts indicate emerging trends in specific research areas, as they have gained significant attention and citations from scholars in recent years (48). Among the top 20 references, the strongest burstiness was caused by the paper, “Cardiac surgery-associated acute kidney injury: risk factors, pathophysiology and treatment,” written by Wang Y et al. with citation bursts from 2019 to 2023 (4). The reference with the second strongest citation burstiness was titled “Foreword,” published in *Kidney International Supplement* by Eckardt KU with the citation burstiness from 2015–2017 (41). Based on the primary research topics of references exhibiting notable bursts in citations (Table 3), biomarkers prediction, risk factors, pathophysiology and treatment are the principal topics at present in the investigation of CSA-AKI. By making use of keywords and references with citation bursts, we are able to quickly grasp the distribution and evolution of hotspots in the research field of CSA-AKI. Table 4 shows the high-frequency keywords, namely, cardiac surgery, acute kidney injury, risk factors, biomarkers and prediction models, highlight the growing emphasis on identifying high-risk patients preoperatively. Clinicians can use these insights to implement targeted interventions, such as optimizing hemodynamics or avoiding nephrotoxic medications, to reduce AKI incidence. The surge in studies on biomarkers like neutrophil gelatinase-associated lipocalin (NGAL) underscores their potential to guide early diagnosis and personalized treatment. According to the trend topic analysis (Figure 9B), the main keywords were biomarker, acute kidney injury, cardiac surgery, intensive care unit, postoperative, prediction model, etc. Machine learning (ML) and prediction models have appeared frequently in the past three years (2021–2023). The emergence of prediction models reflects the clinical need for tools that integrate intraoperative data (e.g., hypotension, fluid balance) and patient-specific factors to predict AKI risk in real-time. Clinicians can use these models to optimize anesthetic management and postoperative care. The keyword cluster “chronic kidney disease” reflects the long-term burden of CSA-AKI. Clinicians should implement follow-up protocols for AKI survivors to detect and manage chronic kidney disease progression.

The integration of ML models into clinical workflows holds significant potential for improving CSA-AKI management. As highlighted in our study, models such as XGBoost and Random Forest integrate dynamic intraoperative variables (e.g., hypotension episodes, cardiopulmonary bypass duration) and biomarkers (e.g., NGAL, angiopoietin-2) to predict AKI risk in real time (18, 49, 50). For instance, the “Detect-A(KI)” tool (51) demonstrates how ML algorithms can process perioperative data streams to generate immediate risk scores.

ML models can be embedded within electronic health record (EHR) systems to trigger alerts for clinicians when high-risk patients are identified. This enables proactive interventions, such as hemodynamic optimization (e.g., maintaining mean arterial pressure >65 mmHg) or avoiding nephrotoxic agents. As demonstrated by Zarbock et al. (2021) and Meersch et al. (2017), early identification of high-risk patients–whether through biomarkers or predictive algorithms–enables targeted interventions such as hemodynamic optimization (e.g., MAP >65 mmHg) and avoidance of nephrotoxic agents, significantly reducing AKI incidence (3, 24). Integrating such risk stratification tools into EHR systems via ML models could further standardize and expedite these interventions. High-risk patients flagged by ML models could receive tailored care bundles, including early nephrology consultation, goal-directed fluid therapy, or biomarker-guided KDIGO protocols (3, 38). For example, the PrevAKI trial (3) demonstrated that biomarker-driven interventions reduced the incidence of moderate-to-severe AKI by 10% in high-risk cohorts.

*Translating Predictions into Clinical Action*: While ML models achieve high accuracy (AUC 0.82–0.94), their clinical utility hinges on actionable outputs. *Risk Stratification*: Models categorize patients into low-, moderate-, and high-risk tiers. High-risk patients (e.g., predicted probability >30% or clinical risk scores) could be prioritized for intensive monitoring or preventive measures, such as remote ischemic preconditioning (23) or amino acid infusions (40). *Dynamic Adjustments*: Intraoperative hypotension is strongly associated with postoperative renal injury, and prolonged exposure to mean arterial pressure <65 mmHg may prompt interventions such as vasopressor titration or fluid resuscitation to mitigate renal hypoperfusion (37, 52). *Postoperative Surveillance*: Post-surgery, ML predictions can guide follow-up strategies. Patients with AKI are at heightened risk for adverse outcomes, including increased mortality and healthcare costs, underscoring the need for vigilant monitoring of renal function during hospitalization (47). Use validated ML models (e.g., XGBoost) or biomarker panels (TIMP-2/IGFBP7) to identify high-risk patients preoperatively. Allocate ICU resources (e.g., continuous renal replacement therapy readiness) for these cohorts. Adopt hemodynamic protocols targeting MAP >65 mmHg (22) and limit red blood cell transfusions (46) to reduce renal ischemia. Consider RIPC (23) in valve/CABG surgeries. Implement KDIGO-based AKI care bundles (e.g., nephrotoxin avoidance, fluid balance monitoring) for all CSA-AKI patients.

Our study highlights the rapid integration of ML into CSA-AKI research, particularly through predictive models like XGBoost and Random Forest. These models outperform traditional risk scores by dynamically integrating perioperative variables (e.g., intraoperative hypotension, cardiopulmonary bypass duration) and biomarkers (e.g., NGAL, angiopoietin-2) to achieve high predictive accuracy (AUCs: 0.82–0.94) (18, 49, 50). Variability in AKI criteria (e.g., AKIN definitions based on serum creatinine and urine output) across studies may complicate clinical outcomes analysis and research validation (19, 29). Most studies remain retrospective or single-center (50, 51). The multicenter study by Demirjian et al. (53) successfully validated the predictive model, but the slight decline in AUC observed in the external cohort suggests that disparities in institutional data practices may impact model generalizability. Enhancing trust in predictive models through rigorous external validation in multicenter cohorts, as demonstrated in studies leveraging routine clinical parameters for AKI prediction (53). Heterogeneous data formats and interoperability issues complicate ML deployment. Standardizing data collection (e.g., FHIR protocols) and developing EHR-embedded decision support tools are essential. ML predictions must complement, not replace, clinical judgment. Protocols should mandate clinician oversight, particularly for high-stakes decisions (e.g., dialysis initiation).

### Future research directions

Our findings align with key recommendations from the KDIGO (21) and ADQI (38) guidelines, particularly in advocating for early biomarker use and hemodynamic optimization. However, bibliometric trends reveal understudied areas. While ML tools show promise, most studies lack external validation across diverse populations (24). Future trials should prioritize generalizability. No RCTs have yet validated ML models’ efficacy in reducing CSA-AKI incidence. We propose trials comparing ML-guided care bundles (e.g., hypotension avoidance, RIPC) versus standard protocols.

### Advantages and limitations

This research holds several distinctive advantages. First, we conducted a systematic bibliometric analysis of CSA-AKI research, which might offer thorough assistance to researchers who follow relevant research. Second, we used four bibliometric tools to perform this investigation, two of which (CiteSpace and VOSviewer) have been widely used in the scientometric field (54). Thus, our data analysis is likely objective. Finally, in contrast to traditional reviews, bibliometric analysis offers a more profound understanding of the hotspots and frontiers. This study also has several limitations. First, data were retrieved only from the WoSCC database in this review; it did not include information from other databases, which might have excluded some important studies. However, WoSCC’s coverage of high-impact journals (e.g., *Critical Care*, *Journal of the American Society of Nephrology*) ensures representation of seminal CSA-AKI research. To validate completeness, we cross-referenced key references identified in WoSCC with PubMed and Scopus, confirming minimal discrepancies. Future studies could benefit from multi-database integration, but for this analysis, WoSCC provided sufficient breadth and depth for robust trend identification. Second, our analysis excluded non-English publications, which may underrepresent research from non-English-speaking regions (e.g., China, Japan, Europe). While this ensured consistency in data extraction and tool compatibility, it limits the capture of region-specific clinical practices or risk factors.

## Conclusion

This decade-long bibliometric analysis delineates the evolving landscape of CSA-AKI research through 4,474 publications from 94 countries. The U.S. and China dominate research output, collectively contributing 45.5% of publications. Fudan University emerges as the most productive institution. Despite active partnerships among leading nations (U.S., China, Germany, Italy), limited engagement persists between high-output institutions (e.g., Fudan University) and international hubs (e.g., Yale University), underscoring urgent needs for collaborative consortia. Seminal works by Rinaldo Bellomo (most co-cited author) and John A. Kellum (highest H-index) established diagnostic criteria, risk stratification frameworks, and biomarker-driven management protocols. Core themes center on pathophysiology, biomarker validation, and prevention strategies, with *Journal of Cardiothoracic and Vascular Anesthesia* and *Critical Care* serving as primary dissemination platforms. Machine learning models (2021–2023) represent a paradigm shift, enabling real-time AKI prediction through integration of dynamic perioperative variables. Keyword trends highlight growing emphasis on personalized prevention, linking intraoperative hemodynamics to post-surgical renal outcomes. By mapping knowledge trajectories and unmet needs, this study equips researchers with actionable insights to advance evidence-based CSA-AKI management. Sustained innovation in predictive analytics and cross-border collaboration will be pivotal in mitigating this costly postoperative complication.

## Data Availability

The original contributions presented in the study are included in the article/Supplementary material. Further inquiries can be directed to the corresponding author.

## Footnotes

^1^ http://bibliometric.com/
