# Supplementary material for: Cardiac surgery-associated acute kidney injury: a decade of research trends and developments
Source: Front Med (Lausanne). 2025 Apr 25;12:1572338. doi: 10.3389/fmed.2025.1572338 (PMC12062005; doi:10.3389/fmed.2025.1572338)
Supplement: Supplementary file 1 [file Data_Sheet_1.docx]

Web of Science (2024-01-03)

| #1 | (TS=(Acute Kidney Injury)) OR TS=(Acute Kidney Injuries)) OR TS=(Kidney Injuries, Acute)) OR TS=(Kidney Injury, Acute)) OR TS=(Acute Renal Injury)) OR TS=(Acute Renal Injuries)) OR TS=(Renal Injuries, Acute)) OR TS=(Renal Injury, Acute)) OR TS=(Renal Insufficiency, Acute)) OR TS=(Acute Renal Insufficiencies)) OR TS=(Renal Insufficiencies, Acute)) OR TS=(Acute Renal Insufficiency)) OR TS=(Kidney Insufficiency, Acute)) OR TS=(Acute Kidney Insufficiencies)) OR TS=(Kidney Insufficiencies, Acute)) OR TS=(Acute Kidney Insufficiency)) OR TS=(Kidney Failure, Acute)) OR TS=(Acute Kidney Failures)) OR TS=(Kidney Failures, Acute)) OR TS=(Acute Renal Failure)) OR TS=(Acute Renal Failures)) OR TS=(Renal Failures, Acute)) OR TS=(Renal Failure, Acute)) OR TS=(Acute Kidney Failure) | 87203 |
| --- | --- | --- |
| #2 | (TS=(Surgery, Cardiac)) OR TS=(Surgery, Heart)) OR TS=(Heart Surgery)) OR TS=(Cardiac Surgery)) OR TS=(Cardiac Surgical Procedures)) OR TS=(Procedure, Cardiac Surgical)) OR TS=(Procedures, Cardiac Surgical)) OR TS=(Surgical Procedure, Cardiac)) OR TS=(Surgical Procedures, Cardiac)) OR TS=(Surgical Procedures, Heart)) OR TS=(Cardiac Surgical Procedure)) OR TS=(Cardiac Surgical Procedure)) OR TS=(Procedure, Heart Surgical)) OR TS=(Procedures, Heart Surgical)) OR TS=(Surgical Procedure, Heart)) OR TS=(Heart Surgical Procedure) | 138002 |
| #3 | #1 AND #2 | 7294 |
